# Mucopolysaccharidosis type IVA and severe hidradenitis suppurativa: A case series

**DOI:** 10.1016/j.jdcr.2025.04.043

**Published:** 2025-06-10

**Authors:** Jorga Fialová, Martina Kojanová, Robert Šáhó, Martin Magner

**Affiliations:** a Department of Dermatovenereology, First Faculty of Medicine, Charles University and General University Hospital, Prague, Czech Republic; b Department of Pediatrics and Inherited Metabolic Disorders, First Faculty of Medicine, Charles University and General University Hospital, Prague, Czech Republic

**Keywords:** adalimumab, brodalumab, hidradenitits suppurativa, morquio syndrome type A, MPS IVA, mucopolysaccharidosis type IVA

## Introduction

Mucopolysaccharidosis type IVA (MPS IVA), also known as Morquio syndrome type A (OMIM #253000) is rare autosomal recessive lysosomal storage disorder resulting from mutations in the GALNS gene, leading to a deficiency in the enzyme of N-acetylglucosamine-6-sulphate sulfatase (GALNS).^1^ The consequent accumulation of keratan sulfate and chondroitin-6-sulfate in various tissues, particularly bone, cartilage, heart valves, and corneas causes skeletal dysplasia including short stature, joint deformities, and other organ involvement. The disease is also characterized by impaired respiratory function, cardiac valve abnormalities, spinal cord compressions, dental issues, impaired vision, and hearing loss, as well as hepatosplenomegaly, with normal cognitive development.^1^, ^2^, ^3^, ^4^ The birth prevalence of Morquio A ranges from 1 per 71,000 to 1 per 179,000 across multiple countries.^5^ Enzyme replacement therapy with elosulfase alfa is the standard first-line treatment for MPS IVA and can help address some of systemic manifestations. However, life expectancy remains reduced, as cardiorespiratory or neurological complications are often the leading cause of mortality.^6^ Hidradenitis suppurativa (HS) is a chronic inflammatory skin disease characterized by painful recurrent abscesses, sinus tracts, and scarring, typically occurring in the intertriginous areas, such as the axillae, groin, and buttocks. The pathophysiology involves follicular occlusion and a dysregulated immune response, particularly involving pro-inflammatory cytokines such as IL-17, TNF-α, and IL-1β. Although the precise genetic and environmental triggers of HS remain unclear, mutations in genes associated with inflammation, such as the γ-secretase complex (PSENEN, PSEN1, NCSTN), have been implicated.^7^, ^8^, ^9^ HS has a prevalence of 0.7% to 1.2% in the general population and is often associated with comorbidities like obesity, metabolic syndrome, type II diabetes, depression, and inflammatory bowel disease.^7^^,^^8^ The treatment of HS includes topical, systemic, and surgical approaches, with biologic therapy being the preferred option for moderate-to-severe cases. Adalimumab (ADA) (TNF-α inhibitor) remains the first-line biologic therapy, given its strongest clinical evidence and regulatory approval. Secukinumab (IL-17A inhibitor) and infliximab (TNF-α inhibitor) are also effective, particularly in ADA non-responders.^10^ Brodalumab (BRO) (IL-17RA inhibitor) has shown promising results in refractory HS, with a 100% HiSCR response at week 12 in an open-label study, though further research is needed.^11^ The expanding range of biologic therapies offers new hope for patients with HS, with personalized treatment based on disease severity and response to previous therapies. This case series reports 3 patients with MPS IVA who developed severe HS, a condition not previously documented in lysosomal storage diseases. We aim to explore the relationship between these conditions and assess the effectiveness of biological therapies in managing severe HS in this patiens population.

## Case descriptions

We present three MPS IVA patients who developed severe HS, which was not previously reported in the context of lysosomal storage disorders. Table I summarizes the main clinical characteristics of the patients.

**Table I:** Main clinical characteristics of 3 patients with mucopolysaccharidosis type IVA and HS

	Patient 1	Patient 2	Patient 3
Current age (y), sex (M/F)	32, F	28, M	24, M
Current height (cm)/weight(kg)	105/36	112/45	110/46
Current BMI (kg/m^2^)	32.65	35.87	38.02
Age at diagnosis of MPS IVA (y)	4	9	5
*GALNS* haplotype (allele 1/allele 2)	p.Arg386Cys/p.Arg386Cys	p.Gly96Cys/p.Arg386Cys	p.Gly96Cys/p.Arg386Cys
Initial clinical signs or symptoms	Pes planus, genu valgum, pectus carinatum	Delayed psychomotor development, macrocephaly, progressive skeletal changes and muscle weakness, and recurrent mesotitis	Progressive muscle weakness, gait disturbance, joint disability (upper and lower limbs), recurrent bronchitis
Current symptoms	Short stature, spinal deformity, significant hip impairment, sternal protrusion, perceptive hypoacusis, restrictive lung disorder, hepatomegaly	Short stature, bilateral perceptual hearing loss, gibbus, sternal protrusion, limited elbow mobility, head tilt, obstructive lung disorder, BiPAP at night	Short stature, sternal protrusion, muscle weakness, wrist and ankle hypotonia, genu valgum, head tilt, obstructive lung disorder, BiPAP at night
Medical interventions	Bilateral wedge resections of the knee joints	Numerous AT, hip and tibial surgeries, cervical spinal cord decompression	AT, surgery on the lower extremities and cervical spine
Age at diagnosis of HS (y)	20	22	13
Smoking history (Y/N)	N	N	N
Family history of HS (Y/N)	N	Y	Y
Localization of HS	Armpits, genitofemoral area, nuchal fold	Armpits, genital area, nuchal fold, chest	Armpits, genitofemoral area, nuchal, cubital, back folds
Current therapy for HS, dose (mg/wk), length of therapy (mo)	ADA, 40 mg/wk, 38	ADA, 40 mg/wk, 5	BRO, 210 mg/wk, 15
Previous medication for HS	DOX	DOX	CLI, DOX, RIF, TMP/SMX, ADA
IHS4 before current biologic therapy	22	43	76
IHS4 after 12 wk of current therapy	2	10	30
IHS4 after 12 mo of current therapy	0	NA	30

Patients 2 and 3 are siblings.

*ADA*, Adalimumab; *AT*, adenectomy; *BiPAP*, bilevel positive airway pressure; *BRO*, brodalumab; *CLI*, clindamycin; *COPD*, chronic obstructive pulmonary disease; *DOX*, doxycycline; *HS*,hidradenitis suppurativa; *IHS4*, International Hidradenitis Suppurativa Score System (the number of nodules [multiplied by 1] plus the number of abscesses [multiplied by 2] plus the number of draining tunnels [multiplied by 4]; a total score of 3 or less = mild, 4-10 = moderate, 11 or higher = severe disease); *RIF*, rifampicin; *TMP/SMX*, trimethoprim/sulfamethoxazole.

**Fig 1:**
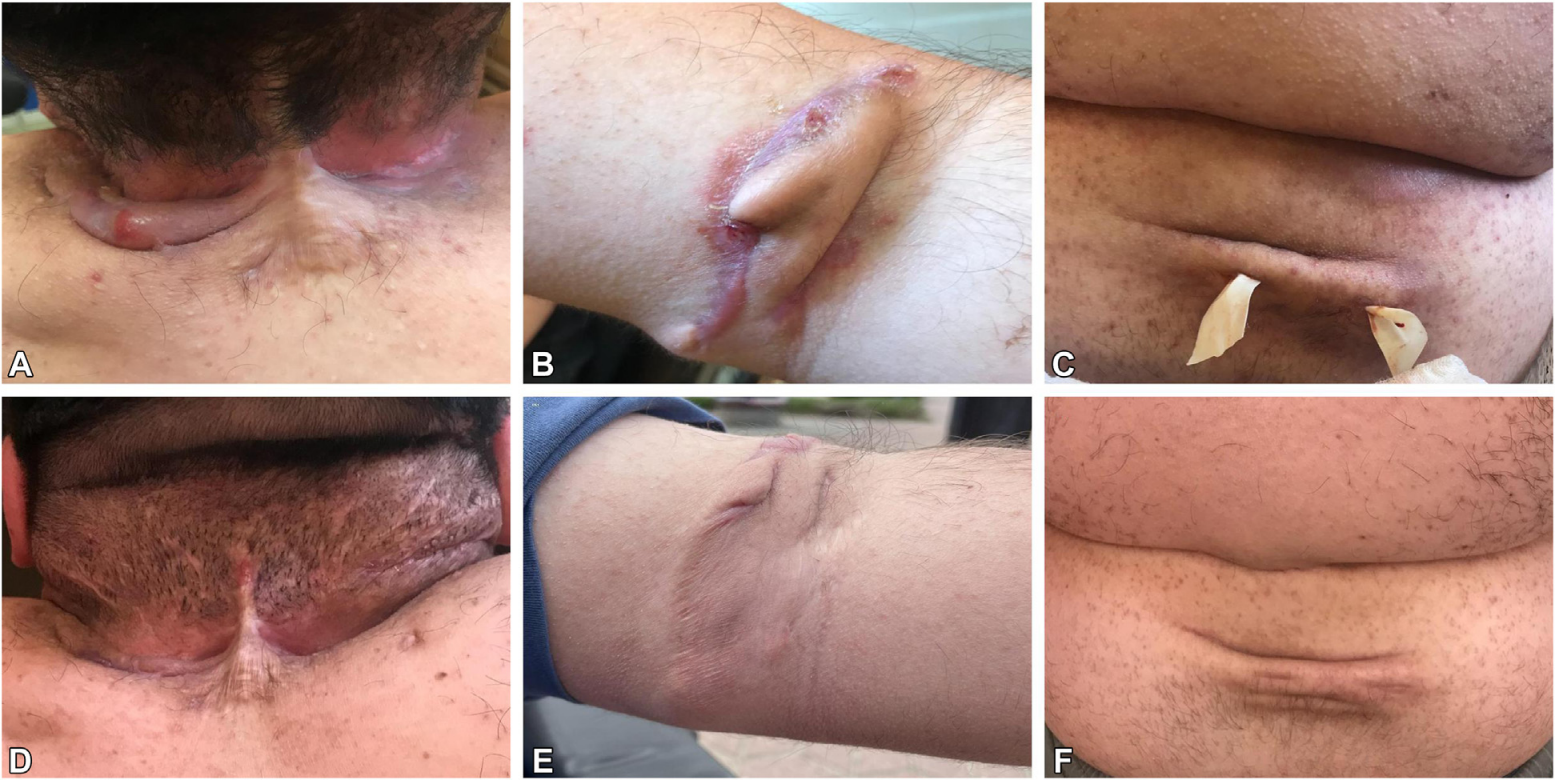
Severe hidradenitis suppurativa in patient 3. Nuchal lesions with chronic inflammation and scarring (**A**); sinus tracts and hypertrophic scarring in the antecubital fossa (**B**); suppurative tunnel with drainage in the lower back region following surgical intervention (**C**). Same anatomical locations after 12 months of treatment with brodalumab, showing marked clinical improvement and scar remodeling (**D-F**).

### Case 1

A 33-year-old female with MPS IVA was diagnosed at the age of 4, initially presenting with pes planus, genu valgum, and pectus carinatum. Over the years, she developed significant skeletal complications, including short stature, spinal deformity, and severe hip impairment, alongside perceptive hypoacusis, restrictive lung disease, and hepatomegaly. By the age of 20, she began experiencing recurrent painful nodules and abscesses localized to the axillary and genitofemoral regions, later extending to the nuchal fold. She had no family history of HS. Before starting biological therapy, she was treated with doxycycline for 1 month, which had to be discontinued due to intolerance. Given the severity of her disease, she was started on ADA at 40 mg per week in 2020, leading to significant improvement. Her IHS4 score decreased from 22 to 2 after 12 weeks of treatment, with complete resolution of active lesions after 12 months. She is currently still on ADA, with practically no active disease.

### Case 2

A 29-year-old male, the sibling of patient 3, was diagnosed with MPS IVA at the age of 9. His initial symptoms included delayed psychomotor development, macrocephaly, progressive skeletal changes, and muscle weakness. Currently, he presents with short stature, bilateral perceptual hearing loss, gibbus, sternal protrusion, limited elbow mobility, head tilt, and obstructive lung disorder, requiring bilevel positive airway pressure at night. He developed HS at the age of 22, with lesions affecting the armpits, genital area, nuchal fold, and chest. Treatment with ADA at 40 mg per week led to a significant reduction in inflammatory lesions. After 12 weeks of therapy, his clinical condition improved notably, with only two inflammatory nodules remaining in the axillae. This improvement has been sustained, and he continues ADA therapy without any changes or reported adverse events.

### Case 3

A 26-year-old male, the sibling of patient 2, was diagnosed with MPS IVA at the age of 5. He initially presented with progressive muscle weakness, gait disturbance, joint disability, and recurrent bronchitis. Currently, he exhibits short stature, sternal protrusion, muscle weakness, wrist and ankle hypotonia, genu valgum, head tilt, and obstructive lung disorder, requiring bilevel positive airway pressure at night. He developed HS at the age of 13, with lesions affecting the armpits, genitofemoral area, nuchal, cubital, and back folds. After failing multiple treatments, including antibiotics and ADA, he was started on BRO at 210 mg per week. After 3 months of therapy, he showed significant improvement, which persisted after 12 months. However, after 2.5 years of therapy, his condition began to worsen, with increasing inflammatory parameters. Due to this deterioration, a change in therapy is currently planned.

## Discussion

This case series reports the rare co-occurrence of MPS IVA and severe HS. Two patients are siblings, and all 3 are paraplegic. The lesions in our patients appeared in typical HS areas, including the axillary, genitofemoral, and gluteal regions. Additionally, nuchal manifestations were observed in all 3 patients, where permanent friction occurs due to head tilt. One patient also developed lesions on the back and in the elbow pit, where a persistent skin fold is present (Fig 1). These findings suggest that mechanical factors, such as chronic pressure and friction from musculoskeletal deformities, may contribute to HS lesion development in patients with MPS IVA. Although this association is rare, biological therapies targeting inflammatory cytokines, such as ADA and BRO, resulted in significant improvements in HS severity. Two patients were successfully treated with ADA and have remained on therapy for 58 and 25 months, respectively, with sustained remission and no significant disease recurrence. The third patient did not respond to several months of antibiotic therapy, surgical procedures, or ADA treatment. He exhibited significantly elevated inflammatory markers, indicating severe and refractory disease. Subsequent treatment with BRO led to gradual clinical improvement, with a significant reduction in inflammatory lesions and markers after 12 months. However, after 30 months of therapy, his condition began to deteriorate. Notably, during the biological therapy, he experienced significant weight gain, which may have contributed to the decreased clinical response. Due to this deterioration, a change in therapy is currently planned. The pathophysiological link between MPS IVA and HS remains unclear, but both conditions involve inflammatory mechanisms that may contribute to their co-occurrence. While no direct genetic or molecular connection has been established, several pathophysiological mechanisms suggest a shared inflammatory and metabolic pathway. One key consideration is the role of Notch signaling in HS. Genetic mutations affecting the γ-secretase complex lead to Notch dysfunction, which plays a central role in HS pathogenesis by impairing follicular differentiation and promoting chronic inflammation. Although Notch pathway dysregulation has not been explicitly linked to GALNS deficiency in MPS IVA, lysosomal dysfunction caused by glycosaminoglycan accumulation could theoretically interfere with Notch receptor processing and degradation, indirectly affecting its signaling activity. Additionally, MPS IVA is associated with chronic systemic inflammation, which could exacerbate the autoimmune and autoinflammatory features of HS. HS is driven by elevated levels of IL-1β, IL-17, IL-23, and TNF-α, cytokines that are also upregulated in systemic inflammatory conditions. Given that patients with MPS IVA exhibit persistent immune activation, this chronic inflammatory milieu may predispose them to HS or worsen its severity. Another contributing factor is microbial dysbiosis. Although HS is not a classical infectious disease, studies indicate that alterations in skin microbiota contribute to chronic inflammation. Patients with MPS IVA may have altered immune responses due to their underlying metabolic disorder, potentially facilitating bacterial colonization and follicular occlusion, thereby triggering HS-like lesions. Lastly, obesity and metabolic factors are well-established contributors to HS. Patients with MPS IVA often have reduced mobility and an increased BMI, both known risk factors for HS. Obesity-driven inflammation, particularly via elevated resistin, chemerin, and leukotriene B4, may further amplify immune dysregulation and keratinocyte hyperproliferation, exacerbating HS symptoms. Understanding these interactions could lead to new therapeutic avenues and improve treatment strategies for patients with MPS IVA and HS. The success of biological therapies in these patients highlights a promising approach for HS management in rare, complex conditions like MPS IVA. Future studies should explore the underlying mechanisms of this association and evaluate the long-term efficacy of biological treatments for HS in patients with rare lysosomal storage diseases.

## Conflicts of interest

None disclosed.
